# One-step endoscopic ultrasound-guided pancreatic duct drainage using a 7-Fr ultra-tapered plastic stent and a novel 0.035-inch guidewire

**DOI:** 10.1055/a-2452-5180

**Published:** 2024-12-10

**Authors:** Takashi Sasaki, Yoichiro Sato, Yuri Maegawa, Takeshi Okamoto, Naoki Sasahira

**Affiliations:** 1 Department of Hepato-Biliary-Pancreatic Medicine, Cancer Institute Hospital, Japanese Foundation for Cancer Research, Tokyo, Japan

An 84-year-old woman with ampullary carcinoma was referred to our hospital ( Fig. 1 ). Robotic-assisted pancreaticoduodenectomy was planned, but only choledochojejunostomy and cholecystectomy were performed because para-aortic lymph node metastasis was detected intraoperatively. The patient subsequently experienced repeated episodes of pancreatitis, and pancreatic duct drainage was planned.

**Fig. 1:**
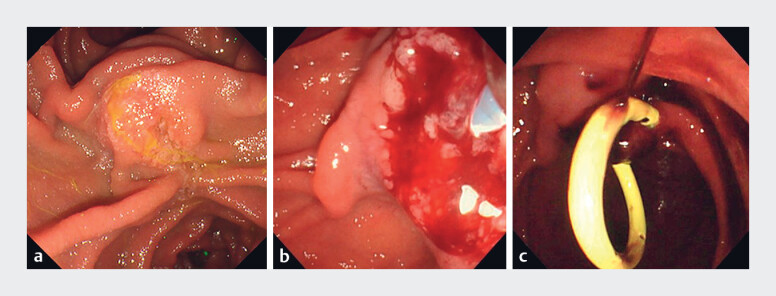
Endoscopic views. **a, b** The ampullary carcinoma. **c** A double-pigtail biliary stent was inserted preoperatively.

Endoscopic ultrasound (EUS)-guided transmural drainage rather than transpapillary drainage was selected based on concerns of further tumor invasion around the papilla in the future. However, computed tomography revealed ascites between the stomach and pancreas ( Fig. 2 ). One-step EUS-guided pancreatic duct drainage (EUS-PDD) without tract dilation was therefore performed ( Video 1 ).

**Fig. 2:**
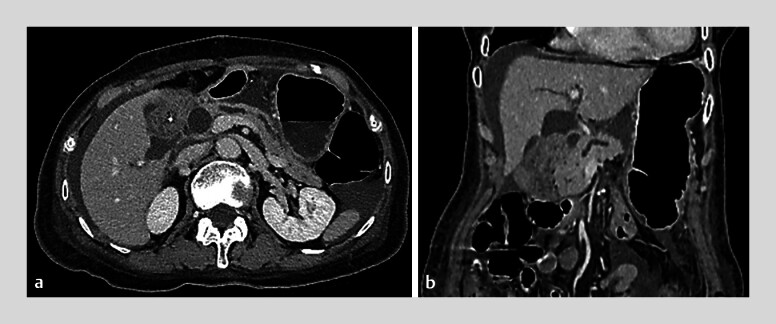
Computed tomography revealed ascites between the stomach and pancreas. The main pancreatic duct was also dilated due to obstruction caused by the ampullary carcinoma. **a** Axial view. **b** coronal view.

One-step endoscopic ultrasound-guided pancreatic duct drainage without tract dilation.Video 1

Fluoroscopy of the main pancreatic duct was achieved after transgastric puncture using a 19-gauge needle. A 0.035-inch guidewire (Capella; Japan Lifeline Co., Ltd, Tokyo, Japan) ( Fig. 3 ) was inserted through the needle into the main pancreatic duct. A 7-Fr ultra-tapered plastic stent (Crane stent; SB-Kawasumi Laboratories, Kanagawa, Japan) ( Fig. 4 ) was then successfully inserted without any tract dilation, with mild resistance.

**Fig. 3:**
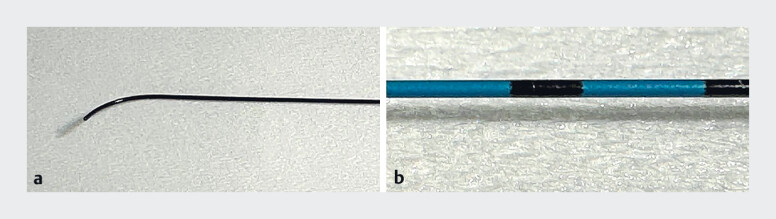
The novel guidewire (Capella; Japan Lifeline Co., Ltd, Tokyo, Japan). **a** This 0.035-inch guidewire could be used like a 0.025-inch guidewire, passing through a 19-gauge needle and a 7-Fr stent delivery system without resistance. **b** The tip of the guidewire is spray-coated to prevent the wire coating from peeling off during guidewire manipulation through a needle.

**Fig. 4:**
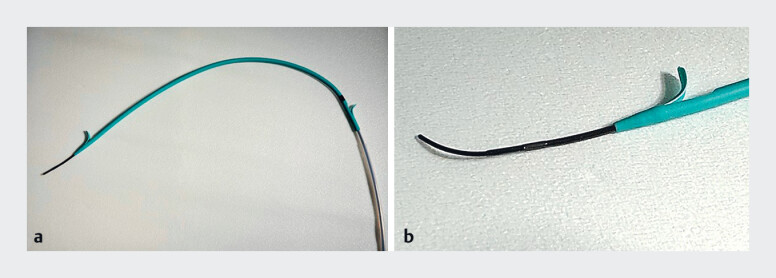
The 7-Fr plastic stent. **a** The ultra-tapered plastic stent system (Crane stent; SB-Kawasumi Laboratories, Kanagawa, Japan) was designed for use with a 0.025-inch guidewire. The stent tip is 4.7 Fr and the tip of the inner catheter is 2.5 Fr. **b** The smooth transition from the 0.035-inch guidewire (Capella) to the 7-Fr ultra-tapered plastic stent (Crane stent) minimizes resistance during stent insertion.

Peritonitis due to pancreatic fluid leakage is a serious complication of EUS-PDD 1 . However, the risk may be reduced if the stent can be inserted without tract dilation. Various novel tapered plastic stents have recently been reported, but they still require tract dilation when used in EUS-guided interventions 2 3 . The plastic stent used in the current case has an ultrafine delivery system, and its use in one-step EUS-guided hepaticogastrostomy has been reported 4 . Ogura et al. reported EUS-PDD using this stent, during which the puncture site was dilated with a standard endoscopic retrograde cholangiopancreatography catheter 5 . The guidewire used in the current case has a 0.035-inch diameter, but could be used like a 0.025-inch guidewire. The guidewire is also rigid, facilitating the transmission of force during unstable maneuvers such as stent advancement during EUS-PDD. The 7-Fr ultra-tapered plastic stent and the rigid 0.035-inch guidewire may be suitable devices for one-step EUS-PDD.

Endoscopy_UCTN_Code_TTT_1AS_2AI

## Notes

Endoscopy E-Videos https://eref.thieme.de/e-videos
*E-Videos* is an open access online section of the journal *Endoscopy* , reporting on interesting cases and new techniques in gastroenterological endoscopy. All papers include a high-quality video and are published with a Creative Commons CC-BY license. Endoscopy E-Videos qualify for HINARI discounts and waivers and eligibility is automatically checked during the submission process. We grant 100% waivers to articles whose corresponding authors are based in Group A countries and 50% waivers to those who are based in Group B countries as classified by Research4Life (see: https://www.research4life.org/access/eligibility/ ). This section has its own submission website at https://mc.manuscriptcentral.com/e-videos .
